# Expression, Purification and Bioactivities Analysis of Recombinant Active Peptide from Shark Liver

**DOI:** 10.3390/md7020258

**Published:** 2009-06-22

**Authors:** Zhengbing Lv, Yu Ou, Qian Li, Wenping Zhang, Boping Ye, Wutong Wu

**Affiliations:** 1 School of Life Science and Technology, China Pharmaceutical University, Nanjing 210009, China; 2 College of Life Sciences, Zhejiang Sci-Tech University, Hangzhou 310018, China

**Keywords:** APSL, prokaryotic expression, bioactivity analysis

## Abstract

The Active Peptide from Shark Liver (APSL) was expressed in *E. coli* BL21 cells. The cDNA encoding APSL protein was obtained from shark regenerated hepatic tissue by RT-PCR, then it was cloned in the pET-28a expression vector. The expressed fusion protein was purified by Ni-IDA affinity chromatography. SDS-PAGE and HPLC analysis showed the purity of the purified fusion protein was more than 98%. The recombinant APSL (rAPSL) was tested for its biological activity both *in vitro*, by its ability to improve the proliferation of SMMC7721 cells, and *in vivo*, by its significant protective effects against acute hepatic injury induced by CCl_4_ and AAP (acetaminophen) in mice. In addition, the rAPSL could decrease the blood glucose concentration of mice with diabetes mellitus induced by alloxan. Paraffin sections of mouse pancreas tissues showed that rAPSL (3 mg/kg) could effectively protect mouse islets from lesions induced by alloxan, which indicated its potential application in theoretical research and industry.

## 1. Introduction

Sharks are one of the most active marine animals. The shark liver, whose weight can account for 75% of the viscera, possesses intense immunoregulation effects and contains some novel bioactive substances. Many researchers have studied the bioactive substances in shark liver [1]. Wu and his research group found an active peptide from shark liver (APSL), related to liver regeneration in *Chiloscyllium plagiosum* and its physicochemical properties and pharmacodynamics have been studied. Due to the low yield of natural APSL from shark liver, its industrial application is limited. This paved the way for obtaining rAPSL by gene engineering methods based on the fact that the cDNA sequence has been obtained by RT-PCR. The overexpression ofrAPSL can make it easy to study the advanced structure and pharmacodynamics mechanism. The use of recombinant APSL is an alternative that could resolve the problem of low availability for industrial applications. Currently, limited information is available on the overexpression of rAPSL, as well as its pharmacological effects. The purpose of this investigation was to overexpress the recombinant APSL (rAPSL) using the gene engineering method, and then rAPSL was purified by SDS-PAGE and HPLC, respectively. Additionally, some *in vitro* and *in vivo* experimental methods were used to identify the pharmacological properties of the purified rAPSL, compared with that of natural APSL.

## 2. Results

### 2.1. cDNA Cloning

We successfully obtained the cDNA fragment of APSL (350 bp) from shark liver by RT-PCR. The expression vector pET28a-APSL was also constructed for overexpress rAPSL in *Escherichia coli*. Six continuous histidines were added to the *N*-terminus of the rAPSL, which greatly accelerated the protein purification process. This work was reported by our group [2].

### 2.2. Expression and Purification of His-Tagged rAPSL

**Figure 1:**
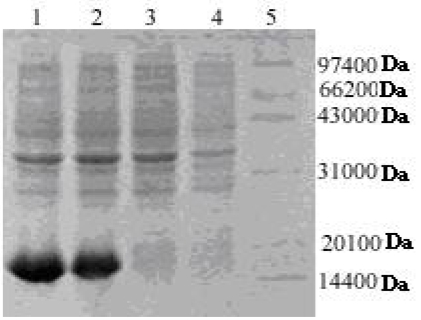
SDS-PAGE analysis of rAPSL. Lane1: Total proteins in the engineering bacteria induced for 5 hours; Lane 2: Total proteins in the engineering bacteria induced for 3 hours; Lane 3: Total proteins in the engineering bacteria induced for 1 hours; Lane 4: Total proteins in the bacteria without objective gene induced for 5 hours; Lane 5: Protein molecular weight marker.

The expression of rAPSL was carried out under different conditions. The expression level was maximum at the 5^th^ hour after induction by IPTG (Figure 1) with a yield of inclusion bodies > 38%.

Nickel metal affinity resin columns were used for single-step purification of rAPSL. The purity of rAPSL was examined by SDS-PAGE and HPLC, and the purity of the fusion protein reached more than 98% by HPLC.

### 2.3. The Study of APSL Bioactivity

The SMMC7721 cell line is generally used as a common method to examine the bioactivities of hepatic stimulator substances (HSS) and similar ones. Our present results showed that 50 μg/mL rAPSL could stimulate the proliferation of SMMC7721 cells, and the potency of the stimulative effect was 1.25 times more than that observed in control wells (Table 1).

Forty-eight hours after administration of CCl_4_ (20 ml/kg body wt), the serum levels of AST and ALT were significantly elevated, from 60.88 ± 10.11 to 164.49 ± 27.16, while the ALT levels were elevated from 31.17 ± 10.53 to 111.4 ± 65.05. rAPSL administrated by the intraperitoneal route (3.0 mg/kg body wt and 1.0 mg/kg body wt) can markedly weaken the higher levels of serum AST and ALT induced by CCl_4_ (Table 2).

After administration of rAPSL at concentrations of 3.0 mg/kg body wt and 1.0 mg/kg body wt, the serum AST levels were decreased by 40.4% and 18.1%, respectively; while the ALT levels of serum were decreased by 44.2% and 33.6%, respectively, compared with the respective serum AST and ALT levels in the model group.

**Figure 2:**
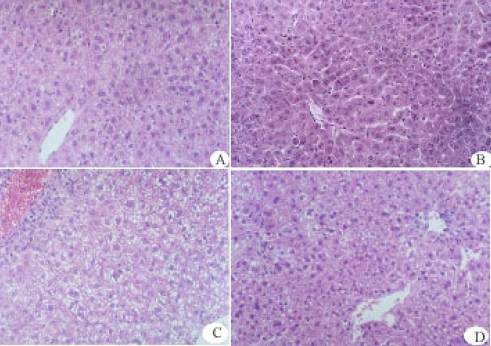
Effect of rAPSL on liver lesions induced by CCl_4_ (× 200 H.E). A: Control; B: treated with 3.0 mg/kg natural product; C: Model; D: treated with 3.0 mg/kg rAPSL.

The histopathologic examination revealed that the cytoplasm of hepatocytes in injured mice liver induced by CCl_4_ was loose. The hepatocytes were markedly edematous, and hepatic lobules and portal area were significantly infiltrated (Figure 2C) compared with the normal hepatocytes (Figure 2A). Intraperitoneal administration of rAPSL (3.0 mg/kg body wt) protected the hepatocytes (Figure 2D) from the injury by CCl_4_. Natural APSL also showed these protective effects (Figure 2B).

**Figure 4:**
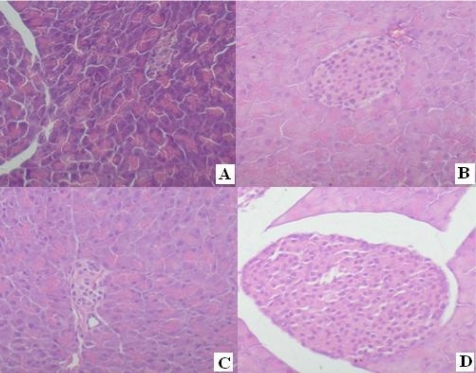
Islet histological findings indicate that alloxan induced islet lesions could be ameliorated by rAPSL in mice (× 200 H.E.). A: Alloxan+Saline; B. Saline alone; C: Alloxan + Insulin; D: rAPSL(3mg/kg) + Alloxan.

The rAPSL can also weaken the elevation levels of serum ALT induced by AAP (Table 3). Compared with the ALT level of the model group, those level of serum ALT in the groups treated with rAPSL (3.0 mg/kg body wt and 1.0 mg/kg body wt) were decreased by 61.5% and 44.1%, respectively. The histopathologic examination also showed that the rAPSL can protect the mice liver against the acute hepatic injury induced by AAP (Figure 4).

The levels of fasting blood glucose (FBG) before and after alloxan administration are shown in Table 4. The FBG level was increased after the induction of diabetes mellitus, which was above 340% (*P* < 0.001 vs. control) at the first week, and then maintained at about 20 mmol/L after continuous administration of alloxan in the fourth weeks. However, the FBG levels were notably decreased to 11.44 ± 6.03 mmol/L (*P* < 0.001 vs. model) after continuous administration of rAPSL (3.0 mg/kg body wt) for three weeks, indicating that rAPSL has the protective effects against the diabetes mellitus in mice induced by alloxan by improving the glucose metabolic disturbance in diabetic mice. Paraffin sections of mouse pancreas tissues showed that rAPSL (3 mg/kg) could effectively protect mouse islet from lesions induced by alloxan (Figure 4).

## 3. Discussion

This research revealed that the recombinant APSL from *Chiloscyllium plagiosum* can improve the proliferation of the SMMC7721 cells as an ALR (augmenter of liver regeneration). However, the results of sequence BLAST between APSL and ALR (including human-ALR and Rat-ALR) showed that the sequence of APSL was significantly different from those of ALR reported by Hagiya [3] and Qiu [4], respectively. No other similar proteins were found. Based on the fact, we regarded it as a novel active protein. Then the cDNA fragment of APSL was obtained in our laboratory by designing the degenerated primer according to the *N*-terminus sequence of APSL instead of the primer of ALR reported by Hagiya [3].

Because the natural APSL in shark liver is very poor, as well as the mRNA of natural APSL, Qiu had found that the mRNA expression of hepatic stimulatory substances in rats increased significantly 12 h after partial hepatectomy and reached the peak 24 h after the surgery [5]. By establishing the partial hepatectomy model to increase the abundance of the APSL mRNA, we successfully cloned the cDNA fragment of APSL from the regenerated hepatic tissues 24 h after partial hepatectomy in shark by RT-PCR.

The BLAST sequence results in GenBank showed that this cDNA fragment was a novel gene including an ORF (open reading frame). The results of alignment between the sequence of translated product from ORF and the sequence of APSL revealed that the *N*-terminus of the translated protein was similar to the natural APSL. The molecular weight of the translated protein was also parallel to that of the natural APSL. Our present results showed that the recombinant protein has bioactivities similar to the natural product both *in vivo* and *in vitro*. From all these studies, we proposed that the cDNA fragment was the sequence of the natural APSL.

Both the natural APSL and its recombinant product have special bioactivities. Our present study showed for the first time that both the rAPSL and the natural APSL have the potential protective effects against diabetes mellitus induced in mice by alloxan, which is similar to the effects of cytokines to stimulate the regeneration of liver. The results showed that both the rAPSL and the natural APSL could be protective against the acute hepatic injury induced by AAP or CCl_4_, and no statistical differences were found. The mice with diabetes mellitus induced by alloxan were administrated rAPSL (3.0 mg/kg body wt) for four weeks, then the FBG levels were notably decreased to 11.44 ± 6.03 mmol/L (*P* < 0.001 vs. model). Paraffin sections of mouse pancreas tissues showed that rAPSL (3 mg/kg) could effectively protect mouse from pancreatic injury caused by alloxan. Our previous research findings also show that the natural APSL also displays protective effects in the pancreas and lowers the FBG levels [6]. So rAPSL has good future prospects for the clinical treatment in type II diabetes mellitus. Moreover, we also first reported the different sequence physicochemical characters and bioactivities of APSL, from ALR. At present, our preclinical research on natural APSL has been finished. In our research, the recombinant APSL (rAPSL) can be overexpressed in *E. coli* BL21. The subsequent researches about APSL should provide us with a better understanding of the functions of APSL. This work will be beneficial to basic research and industrial applications.

## 4. Experimental

### 4.1. Materials

*Chiloscyllium plagiosum* was purchased from Nanjing Huiminqiao market. Male ICR mice [certificate No. SCXK (Su) 2002-0015], weighing 18~22 g, were purchased from the Animal Center of Nanjing Medical University. AAP purchased from ICN Biomedicals Inc., was dissolved with saline before use. Alloxan, RNasin, M-MLV, Taq DNA polymerase, dNTPs and pGEM-T Easy Vector were all purchased from Promega. The expression vector pET-28a, the host strain BL21 (DE3) and His-Bind Kit were purchased from Novagen. DNA Recovery Kits were offered by Vitagene. The purity of APSL which was prepared by our laboratory and analyzed by high-performance liquid chromatography (HPLC) was above 98%.

### 4.2 Cloning of the cDNA Fragment of APSL by RT-PCR

Based on the report from Ou [7] about the *N*-terminus sequence of APSL, we designed one degenerate 5′-AT(C)TIGTIGGICCIATC(T)GGIGCIG-3′ primer. The PH (Partial Hepatectomy) of shark liver model was built according to the method reported by Ye [8]. The total RNA was extracted from the regenerated hepatic tissues, and then the reverse transcription PCR was performed with that degenerated primer and Oligod(T)_18_. PCR product was purified with DNA Recovery Kits and ligated with pGEM-T Easy Vector. Recombinant vector was selected by digestion with *Eco*R I and sequencing.

The PCR product (350 bp) was collected, digested with *Nde* I and *Sal* I, and then subcloned into the pET-28a plasmid. The recombinant vector, named pET28a-APSL, was selected for sequencing after PCR analysis and restriction enzyme digestion [2].

### 4.3. Overexpression and Purification of Recombinant APSL (rAPSL)

*E. coli* BL21 (DE3) with the recombinant expression plasmid pET28a-APSL was grown in LB liquid medium containing 50 μg/mL kanamycin at 37 °C in a shaking incubator. When A_600_ was about 0.4, IPTG was added into the medium with a final concentration of 1 mmol/L to induce expression of recombinant APSL, and then incubation continued at 37 °C with continuous agitation. At the 0, 1, 3 and 5 h time points of the induction period, samples were centrifuged, and the protein of interest was analyzed by SDS-PAGE, gel electrophoresis according to the protocol described by Sambrook [9].

For high expression of recombinant APSL, the cells were harvested during the incubation at 37 °C at 0, 1, 3, and 5 h time points after IPTG induction (final concentration of 1 mmol/L), then the cells with optimized expression were harvested by centrifugation and washed with 10 mmol/L Tris-HCl. The precipitate was dissolved with 50 mmol/L Tris-HCl and the cells broken with ultrasound on ice. The solution was centrifuged at 5,000 g for 10 min at 4 °C in order to collect the pellets. The pellets were dissolved into 6 mol/L urea and 1 × binding buffer (5 mmol/L imidazole, 0.5 mol/L NaCl, 20 mmol/L Tris-HCl pH 7.9), and stored at 4°C. The fusion APSL was purified with a His-Bind kit according to the protocol described by Novagen Co. The purified recombinant APSL was washed by turns twice with 10 mmol/L Tris-HCl (pH 7.9) containing 0.3% mercaptoethanol and twice with ddH_2_O containing 0.3% β-mercaptoethanol. The purified protein was analyzed by SDS-PAGE and HPLC (Zorbax 300SB-C_18_), respectively. SDS-PAGE was carried out using 12% resolving gel and 5% stacking gel. The mobile phase A of HPLC was H_2_O and 0.1% TFA, and the mobile phase B was analytical grade CH_3_CN.

### 4.4. Improved Effects of rAPSL to the Proliferation of SMMC7721 Cells

The bioactivity of rAPSL *in vitro* was tested by the MTT (3-(4,5-dimethylthiazol-2-yl)-2, 5-diphen-yltetrazolium bromide) method [10]. According to the results of extensive pretests, 5 × 10^4^ cells/well MTT solution (5 μg/mL) were selected to carry out the test. The protocol of the present MMT study was performed as follows: after SMMC 7721 cells were seeded at 10^4^ cells/well in a 96-well plate, the various concentrations (12.5, 25, 50 μg/mL) of rAPSL and natural APSL were added to the 96 wells. Then the plates were incubated for 30 h at 37 °C in a 5% CO_2_ humidified atmosphere. After the incubation period, 20 μL of MTT solution from a 5 mg/mL stock solution prepared in PBS was added to each well. Plates were returned to the incubator for 6 h. After the 6 h incubation period, the MTT solution was replaced by DMSO. The plates were incubated at 37 °C for 10 min with agitation. MTT conversion was measured using a microplate reader (Bio-Rad, Model 680), reading the absorbance at 570 nm. Cell proliferation (as a percent of the negative control) was evaluated by the absorbance values.

### 4.5. Protective Effects of rAPSL against Mice of Acute Hepatic Injury Induced by CCl_4_ or AAP

Fifty male ICR mice weighing 18~22 g were randomly divided into five groups, including the control group, the model group, the group treated with natural APLS (3.0 mg/kg body wt), and the group treated with rAPSL at high dosage (3.0 mg/kg body wt) and at low dosage (1.0 mg/kg body wt), respectively. All groups were treated with the dose intraperitoneally for 4 days (twice per day). The mice, except the control group, were injected with 0.2% CCl_4_ intraperitoneally half hour after the third administration, CCl_4_ was dissolved in olive oil [11] and injected at a volume of 20 mL/kg. The mice in the control group and model group were injected with 10 mmol/L Tris-HCl. The blood samples were obtained from the medial canthus after the last time administration. Serum alanine aminotransferase (ALT), aspartate aminotransferase (AST) were determined by modified Reitman-Frankel’s method. The liver samples were obtained from the hepatic median lobe after the last time administration and fixed in 10% formalin solution. Stained with hemotoxylin and erosin, the specimens were observed under microscope. With the same method and dosage, we also tested the protective effect of rAPSL against mice with acute hepatic injury induced by acetaminophen (AAP). AAP was dissolved in phosphate buffer saline (PBS, pH 7.4) immediately before each experiment. The dosage of AAP was 200 mg/kg body weight [12].

### 4.6. Protective Effects to the Diabetes Mellitus Mice Induced by Alloxan

Ninety ICR mice, equivalent numbers of females and males, weighing 18~22 g were randomly arranged in six groups, including the control group, the model group, the insulin treated group (6 U/kg body wt), the group treated by natural APLS (3.0 mg/kg body wt), and the group treated by rAPSL at high dosage (3.0 mg/kg body wt) and low dosage (1.0 mg/kg body wt), respectively. Mice in the control group and the model group were treated only with saline (20 ml/kg body wt) [6]. To induce diabetes mellitus, the mice except for the control group were injected with alloxan (70 mg/kg body wt) intravenously 12 h after fasting. Fasting plasma glucose (FPG) levels were measured 3 days after the alloxan administration. Only mice with FPG levels over 11.1 mmol/L were selected for experiments. The FPG levels were measured again at the day of 7^th^, 14^th^, 21^st^ and 28^th^ after the alloxan administration.

### 4.7. Statistical Analysis of the Data

All results were presented as *x̄* ± s, “Student” *t*-test was used for statistical analysis and statistical significance was defined as *P* < 0.05 or *P* < 0.01.

## Figures and Tables

**Figure 3:**
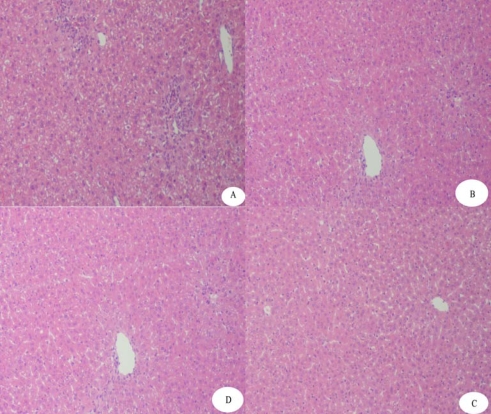
Effect of rAPSL on liver lesions induced by AAP (× 100 H.E). A: Model; B: Control; C: treated with 3.0 mg/kg rAPSL; D: treated with 3.0 mg/kg natural product.

**Table 1:** Effects of rAPSL on the growth of SMMC7721 cells.

Group	Dosage (μg/mL)	OD_570_
Control	-	0.36 ± 0.03
APSL	50	0.65 ± 0.12**
rAPSL	50	0.45 ± 0.02**
	25	0.41 ± 0.04
	12.5	0.40 ± 0.03

** *P* < 0.01 vs control, n = 4, *x̄* ± s.

**Table 2:** Effect of rAPSL on the levels of ALT and AST induced by CCl_4_ in mice.

Group	Dosage mg/kg	AST (OD_505_)	AST (cal’s unit)	ALT (OD_505_)	ALT (cal’s unit)
Control	-	0.141 ± 0.017**	60.88 ± 10.11**	0.087 ± 0.022**	31.17 ± 10.53**
Model	-	0.317 ± 0.046	164.49 ± 27.16	0.256 ± 0.079	111.4 ± 65.05
APSL	3	0.220 ± 0.029**	107.43 ± 16.88**	0.153 ± 0.034**	62.60 ± 21.63**
rAPSL	3	0.203 ± 0.061**	97.79 ± 35.94**	0.152 ± 0.027**	62.12 ± 15.80**
	1	0.266 ± 0.035*	134.71 ± 20.53*	0.177 ± 0.045*	73.96 ± 32.37*

* *P* < 0.05,

** *P* < 0.01 vs model, n = 10, *x̄* ± s

**Table 3:** Effect of rAPSL on the level of ALT induced by AAP in mice.

Group	Dosage (mg/kg)	ALT(OD_505_)	ALT (cal’s unit)
Control	-	0.067 ± 0.016**	21.52 ± 7.94**
Model	-	0.234 ± 0.081	101.17 ± 38.83
APSL	3	0.104 ± 0.022**	39.26 ± 10.63**
rAPSL	3	0.104 ± 0.026**	38.90 ± 12.39**
	1	0.141 ± 0.062*	56.58 ± 29.62*

* *P* < 0.05,

** *P* < 0.01 vs model, n = 10, *x̄* ± s

**Table 4:** Effect of rAPSL on fasting blood glucose level in diabetic mice.

Group	Dosage mg/kg	FPG(mmol/L)			
		First week	Second week	Third week	Forth week
Control	-	6.37 ± 1.10	6.42 ± 1..24	6.5 ± 1.24***	6.74 ± 1.31***
Model	-	HI	HI	22.73 ± 3.65	20.08 ± 5.18▴▴▴
INS	6★	4.25 ± 0.63	4.07 ± 0.73	4.23 ± 1.28***	4.32 ± 1.10***
rAPSL	3	HI	HI	12.28 ± 4.54***	11.44 ± 6.03***
	1	HI	HI	20.21 ± 5.92	18.04 ± 6.71

*** *P* < 0.001 vs model,

▴▴▴ *P* < 0.001 vs control, *x̄* ± s, n = 10~12,

★ u/kg; HI: > 28 mmol/L.
